# Emerging Hand Foot Mouth Disease in Bangladeshi Children- First Report of Rapid Appraisal on Pocket Outbreak: Clinico-epidemiological Perspective Implicating Public Health Emergency

**DOI:** 10.12688/f1000research.15170.3

**Published:** 2019-06-28

**Authors:** Md. Azraf Hossain Khan, Kazi Selim Anwar, A. K. M. Muraduzzaman, Md. Abid Hossain Mollah, S. M. Akhter-ul-Alam, Kazi Munisul Islam, Sheikh Ariful Hoque, Md. Nazrul Islam, Md. Ahasan Ali

**Affiliations:** 1 Department of Dermatology and Venereology, Pabna Medical College and General Hospital, Pabna, 6600, Bangladesh; 2 US-CDC’s GHSA Project, Institute of Epidemiology, Disease Control and Research (IEDCR), Dhaka, 1212, Bangladesh; 3 Department of Virology, Institute of Epidemiology, Disease Control and Research (IEDCR), Dhaka, 1212, Bangladesh; 4 Department of Pediatrics, Ibrahim Medical College & Hospital, Institute of Research & Rehabilitation in Diabetes, Endocrine and Metabolic Disorders (BIRDEM), Dhaka, 1200, Bangladesh; 5 Infectious Disease Division, International Center for Diarrheal Diseases Research, Bangladesh (icddr,b), Dhaka, 1212, Bangladesh; 6 Tissue Culture Laboratory, Centre for Advanced Research in Sciences (CARS), University of Dhaka, Dhaka, 1000, Bangladesh; 7 Microbiology Section, Institute of Public Health (IPH), Mohakhali, Dhaka, 1212, Bangladesh

**Keywords:** Emerging Childhood-HFMD, Bangladesh, Rapid-Appraisal, Pocket-Outbreak

## Abstract

**Background: **Hand, foot and mouth disease (HFMD) is a common contagious disease among children under 5 years, particularly in the Asia-Pacific-region. We report a localized outbreak of childhood HFMD for the first time from Bangladesh, diagnosed only based on clinical features due to lack in laboratory-diagnostic facilities.

**Methods: **Following the World Health Organization’s case-definition, we conducted a rapid-appraisal of HFMD among all of the 143 children attending Pabna Medical College and General Hospital with fever, mouth ulcers and extremity rash. Data were collected between September and November 2017 using a preset syndromic approach and stringent differential diagnostic-protocols.

**Results: **The mean age of children was 2.9±2.3 years. There was a significant difference among the age and sex of children (P=0.98), first sibling being more belonging to middle-income families (62%). Younger children (<5 years) were more likely to suffer with moderate-to-high (38.5°C) fever (P<0.04), painful oral ulcers (P<0.03) and painful/itchy rash (P<0.01). Sex did not differ with other symptoms, but boys had less painful oral ulcers than girls (P<0.04). Fever (63%) and chicken-pox-like-rash (62%) was observed more in mid-October to mid-November than September to mid-October (P<0.01 and P<0.03, respectively). No differences in symptoms (fever, oral ulcers and extremity rash) were observed with precipitation, nor with ambient temperature. Children <5 years (85%) had quicker recovery (within 5 days) than those ≥5 years (69%), (P<0.04), with marginal differences in sex (P<0.05).

**Conclusions: **Our findings highlight potential usefulness in diagnosing HFMD based on clinical parameters, although stringent differential diagnosis remains indispensable, which is particularly applicable for resource-constrained countries lacking appropriate virology/essential laboratories. Since no specific treatment or effective vaccination is available for HFMD, supportive therapy and preventive measures remain the primary methods to circumvent disease-transmission augmented by climate-related factors. Standardized virology laboratory warrants appropriate diagnosis and globally representative multivalent-vaccine deem essential towards preventing HFMD.

## Introduction

Of all commonly occurring febrile illness and rash syndromes ^1^, hand, foot and mouth disease (HFMD) remains the most among young children ^2, 3^. Although this viral infection remains largely contagious ^4, 5^, it is self-limiting and benign. Severe cases reportedly occurs in lower incidences (3.2% to 8.5%) and fatalities are rare ^6– 8^. Starting in the West during the mid-1970’s ^1, 2^ HFMD emerged in the Asia-Pacific region in mid-1990s ^9– 11^ heralding as a major public health hazards ^2, 10^. Epidemiologically, it follows a 2–3 years cyclical pattern ^11^ but may break out anytime ^9^ as has occurred in India (Orissa ^12^ and Calcutta ^13^), bordering with Bangladesh.

With the complaints of mild-to-moderate fever (≥38.5°C ^8^; 101.3°F) childhood HFMD, characteristically manifest with body rashes ^1, 4^, mostly of the knees and buttocks ^4, 14^, augmented by painful oral/buccal ulcers and blisters. Papulo-vesicular rash in the extremities consequently forms pustules ^6^. Most children recover/heal within 7–10 days ^5, 8, 9^. Of the few complications, neuro-respiratory syndromes ^4^ (encephalitis, aseptic meningitis and acute flaccid paralysis) ^3, 4^ occur mainly in younger children; these are rare but seldom fatal ^9, 15^. HFMD is caused by several serotypes of enterovirus A, the most common being enterovirus A71 (EV A71) ^2, 16^ and coxsackievirus A16 (CV-A16) and more recently, also (CV A-6, and CV A-10) ^5, 7, 10^. EV-A71 is associated with a higher proportion of severe illnesses ^2, 16^. Reportedly, these viruses are transmitted ^15, 17^ through direct contact/blister-fluid, droplets, oro-fecally ^16^ and also spread out through contaminated environment, water and food ^18^.

Reportedly, clinical diagnosis of HFMD is usually established depending on physicians’ suspicions ^13, 14^ as the sole diagnostic modality ^12^. The diagnosis is primarily based on history of illness, disease-onset, presenting clinical-features ^1, 6, 19^ and, socio-demographic profile ^12, 14, 20^. Small erythematous maculopapular lesion (1–5 mm) enlarge (3–15 mm) and progress to vesicular eruptions with a prominent erythematous halo ^13, 21^. It is essential to perform stringent differential diagnosis (DD) to distinguish HFMD from a group of diseases. DD includes chickenpox, scabies, measles, erythema multiforme, herpangina, herpetic gingivitis, drug eruption and others ^4, 14, 17, 22^. Laboratory diagnosis is usually not essential ^12, 19, 23^, and has been described by the World Health Organization (WHO) as optional ^1^. Conversely, the sophisticated laboratory tests used for definitive diagnosis (virus isolation, molecular analysis, PCR, genotyping) ^1, 13, 24^ are not available in most resource-constrained countries ^3, 12, 13^ like Bangladesh.

Since there is no specific treatment ^4, 19, 22, 25^ for HFMD, care largely remains palliative ^19^ with antipyretics/analgesics and antihistamines. Topical anesthetics are rarely used for oral ulcers for soothing and comfort. Povidone-iodine used as a mouth wash/topical application that can relief pain. Since no effective vaccine against HFMD-viruses is available ^1, 2, 7^, preventive measures remain the primary method of circumventing HFMD transmission to break infection-chains (droplets, oral-fecal route, and direct contact) ^2, 18^. Effective prevention requires personal hygiene, hand washing ^26^ and a pollution-free environment ^12^ including food and water ^18, 27^. Meteorological variations in precipitation ^8, 9^ and ambient temperature ^20, 28^ often impact on HFMD occurrences ^5^ in the Asia-Pacific region ^5, 9, 10, 15, 17^, along with atmospheric pressure and the relatively higher humidity in summer and early autumn ^18^.

Extracts from extensive reviews, when compared with our intensive observations on upsurge of unusual febrile, rash-associated childhood illnesses between July and August 2017, were indicative of HFMD. A rapid appraisal was therefore, designed as a short-term standardized-surveillance ^1^. Following a pre-set case-definition and syndromic approach (according to the WHO HFMD guidelines ^1^), similar to a study conducted in Thailand ^29 ^ a strategic plan was adopted to conduct this comprehensive study from September to November 2017.

## Methods

### Set up, patients and research design

Utilizing a pre-set syndromic approach based on case-definition following the WHO’s HFMD guidelines ^1^ this rapid appraisal was conducted among all the 143 children attending Pabna Medical College and General Hospital (PMC-GH) between September and November, 2017. PMC-GH is a 250-bed secondary care hospital serving a targeted population of nearly 2.81 million from its 2,371.5 km ^2^ ^30^ catchment area situated in a small poverty-stricken north-western flood-prone plain land on the Ganges Delta basin in Bangladesh.

### Research instruments used

***Clinical diagnostic tool.*** Prepared based on syndromic case-definition following the WHO’s HFMD guidelines ^1^, similar to a prior study conducted in Thailand ^29^. Most of the contents of this tool have been shown in Figure 4 (4 A), showing the algorithm of Clinical diagnosis of HFMD ^1^.

***Clinical case management protocol***. This was prepared incorporating a history of disease, onset, chief complaints and duration of illness, clinical diagnosis and therapeutic intervention. We ascertained clinical outcome by through post-treatment follow-up in the outpatient department of PMC-GH or through cellphone-based enquiry. We performed the clinical diagnosis following WHO guidelines ^1^, predominantly based on three main signs and/or symptoms: fever, oral ulcers and rash in extremities. Fever was graded into moderate-to high (38.5°C) and none-to-low (37-38.4°C), oral ulcers were grouped into three stages- more painful, less painful & painless; and, rashes in extremities into three types: painful and itchy, painless and itchy; and painful but not itchy.

### Pain assessment/scoring tool

Since pain remains subjective in younger children in expressing pain intensity properly, we arbitrarily categorized the pain intensity based on following clinical grounds:

i. Nullifying any history of similar disease/disorders in near past
ii. Facial expression of a child with body rash and/or oral ulcer on touch
iii. Impression and/or opinion of child’s parent/guardian in respective cases
iv. Finally, clinician’s judgements based on history and presented signs/symptoms

### Therapeutic management guideline

A therapeutic guideline was prepared to treat childhood HFMD cases following standard therapeutic plan consisting of: antipyretic/analgesics, antihistamines, anesthetic-cream for topical applications.

### Epidemiological tool

This tool consisted of socio-demographic variables and household (HH) income. We categorized the monthly (mon) income (in Bangladeshi taka: BDT) of child’s family according to World Bank (WB) Data Help Desk 2016 ^31^ as follows:

- Low-income group: HH income of ≤ 6,946/ mon
- Lower-mid income group: HH income: 6,947–27,336/mon
- Upper-mid-income group: HH income:27,337–84,564/mon
- High-income group: HH income of ≥ 84,564 BD/ mon

(Calculated using USD rate: 1US $=84.31 BDT dated 11.06.18)

### Seasonal data collection

Seasonal data on local weather/climate for average temperature and rain precipitation were collected from Pabna Meteorology Department, Bangladesh over the period of September through November 2017. In Bangladesh, early autumn runs from September to mid-October, followed by late autumn/fall from mid-October to mid-November.

All these tools (developed or followed) were duly pre-tested for this rapid-appraisal (small-scale disease surveillance) ^1, 2^.

### Data analysis

Crosschecked data were subjected to Pearson’s chi-squared test, Fisher’s exact test and Spearman correlation analysis using SPSS for Windows v.21, taking P<0.05 as indicating statistical significance (at 95% CI).

### Inclusion criteria/patient enrolment

Any child, irrespective of age and sex, attending PMC-GH between September and November 2017 with suspected HFMD (meeting WHOs ^1^ recommended criteria) were included in this study. Suspected cases having other serious disease/co-morbidities were excluded, although patients were referred to concerned department for proper clinical management.

### Ethical considerations

Following standard procedure of ethical issues ^32^, written informed consent was obtained from the parents of children with suspected HFMD prior to enrolment. We detailed the parents/guardian of all children on the purpose and procedures of this study. We also informed the parents on the lack of risk of harm/damage involved in procedures and did not collect body fluids or other biological samples. We informed the parents that they could remove their child at any stage of the study. Complete privacy and anonymity of clinical data was ensured, including its protected use research purposes only. This study had prior approval through the Ethical Committee of Pabna Medical College and General Hospital, Government of the Peoples’ Republic of Bangladesh (Memo No. 1577, dated: 26/08/2017).

## Results

### Demographic information

The mean (±SD) age of the 143 children was 2.9±2.3 years; 80 (56%) were boys and 63 (44%) were girls. Of the total, 70% were under 5 years old. Age did not differ with sex (P=0.98). Data on HH structure yielded an average size of children’s family as 5.5±6.9 persons/per HH. Of them, 62% having only one (no siblings) and 38% two (first sibling) children, ( Table 1).

**Table 1.** Socio-demographic characteristics and household income of child’s family attending the Pabna Medical College and General Hospital with the complaints of hand, foot and mouth disease (n=143 cases).

Variable	Groups	N (%)
Age	2 months–3 years	78 (54.5)
	3.1–5 Years	32 (22.4)
	>5.1 Years	33 (23.1)
Sex	Male	80 (55.9)
	Female	63 (44.1)
Age vs. sex		
χ ^2^	p=0.98	
Likelihood ratio	p=0.98	
Spearman’s correlation	p >0.87	
Siblings	Child 1	89 (62.2)
	Child 2+	54 (37.8)
Household income *	Low income	21 (14.7)
	Low-mid-income	73 (51.0)
	Upper-mid-income	49 (34.3)
	High income	0
Sibling number vs. household income		
χ ^2^	p <0.01	
Likelihood ratio	p =0.01	
Spearman’s correlation	p<0.01	

*Following World Bank Data Help Desk, 2016 ^33^

Following Word Bank, (2016) standard ^31^ family/HH income-group evidenced that majority families (85%) belonged to middle-income HH/families (34% belonged to upper-middle and 51% to lower-middle income-groups living with a modest HH budget). The rest (14.7%) belonged to low-income groups lived with a tight HH-budget. Notably, HFMD cases were significantly more common among children from mid-income-HHs and among first siblings (P<0.01), ( Table 1).

### Assessment of symptoms

Child’s age was significantly associated with three major clinical signs/symptoms. Younger children (under 5 years old) suffered more (74/91, 81%) with moderate-to-high fever than older children (17/91, 19%; p<.04). Similarly, painful oral ulcers (82/111, 74%) and painful itchy rash in extremities (92/116, 79%) were more common in younger than older children (p<0.03 and p<0.01, respectively). Notably, skin rash in extremities of younger children’s were predominantly more like papulo-vesicular (59/68, 87%) than chicken pox-like (43/75, 57%) lesions (p<0.001). However, sex did not differ with other signs/symptoms except oral ulcers: boys had less painful ulcers (23/32, 72%) than girls (9/32, 28%), (P<0.04), ( Table 2).

**Table 2.** Composite table showing association of HFMD clinical features with age and sex.

Variables	Clinical manifestation							
	Body temperature		Oral ulcers		Rash in extremities		Rash characteristics	
	≥38.5°C (n=91)	37–38.4°C; (n=52)	Painful (n=111)	Painless/less- painful (n=32)	Painful/itchy (n=116)	Painless/less painful (n= 27)	Chicken pox-like (n= 75)	Papulovesicular (n= 68)
Child’s age								
<3 years (n=78)	57	21	54	24	70	08	32	46
≥3 but <5 years (n=32)	17	15	28	4	22	10	19	13
≥5 years (n=33)	17	16	29	4	24	09	24	09
χ2	P<0.04		P<0.03		P<0.01		P<0.01	
Spearman’s correlation	P<0.01		P=0.01		P <0.01		P<0.01	
Sex								
Male (n=80)	52	28	57	23	68	12	44	36
Female (n=63)	39	24	54	9	48	15	31	32
Fisher’s exact test	P>0.73 (2-sided); P>0.42 (1-sided)		P>0.04 (2-sided); P>0.03 (1-sided)		P>0.20 (2-sided); P>0.13(1-sided)		P>0.51 (2-sided); P>0.30(1-sided)	
Spearman’s correlation	P>0.71		P<0.04		P<0.18		P<0.49	

*Mean ± SD = 2.9±2.3.

None of the three major signs/symptoms of HFMD (fever, oral-ulcers/blisters and extremity rash) was associated with seasonal variations except fever and characteristics of rash. Moderate-to high fever (57/91, 63%) was observed more in fall/late-autumn (mid-October through mid-November) than in early autumn (September through mid-October), yielding 37% of cases (34/91), (p<0.01). Similarly, papulovesicular rashes were more common in fall (42/68, 62%) than in early autumn (26/68, 38%) (P<0.03) ( Table 3).

**Table 3.** Composite table showing association of HFMD clinical features with season/local climate.

Variables	Clinical manifestation							
	Body temperature		Oral ulcers ^$^		Rash in extremities ^$^		Rash characteristics	
	38.5°C (n=91)	37–38.4° C (n=52)	Painful (n=111)	Painless/less- painful (n=32)	Painful (n=116)	Painless/less painful (n=27)	Chicken pox like (n=75)	Papulo- vesicular (n=68)
**Seasons**								
September-mid- October (n=42)	34	8	33	9	36	6	16	26
Mid-October-mid- November (n=101)	57	44	78	23	80	21	59	42
Fisher’s exact test	P<0.01 (2-sided) & <0.01 (1-sided)		p>1.0 (2-sided) & 0.53 (1-sided)		p>0.48 (2-sided) & 0.26 (1-sided)		p>0.03 (2-sided) & 0<0.02 (1-side)	
Spearman’s correlation test	p< 0.01		p>0.86		p>0.37		p<0.03	
Average rainfall on admittance								
0.0 mm (n= 107)	67	40	85	22	86	21	56	51
01.7 mm (n= 22)	15	7	17	5	19	3	9	13
>20.1 mm (n= 14)	9	5	9	5	11	3	10	4
*χ* ^2^-Chi-square test	p =0.88		p>0.44		p <0.78		p <0.20	
Spearman’s correlation test	p >0.70		p =0.32		p <0.76		p <0.77	
Ambient temperature on admittance								
24.4–29.9°C (n= 22) ^a^	11	11	20	2	16	6	14	8
≥30°C (n=121) ^b^	80	41	91	30	100	21	61	60
Fisher’s exact test	p>0.16 (2-sided) and 0.12 (1-sided)		p>0.16 (2-sided) & >0.08 (1-sided)		p>0.37 (2-sided) & 0.21(1-sided)		p>0.35 (2-sided) & 0.18 (1-sided)	
Spearman’s correlation test	p< 0.15		p>0.11		p>0.28		p>0.26	

^a^ Comparatively lower temperature: Arbitrarily set cut-off values of lower temperature (on average). ^b^ Comparatively higher temperature: Arbitrarily set cut-off values of higher temperature (on average). The three major sign/symptoms of HFMD among these children were more prevalent on those days when the rain precipitation was recorded 0.0 mm, in our outbreak areas. Rain had no significant impact on any of the three major sign/symptoms, unlike on dry days with no rainfall (0.0 mm). Similarly, all major sign/symptoms prevailed more in hot and humid days when the ambient temperature was recorded at ≥30°C (up to a maximum of 36.2°C), with no significant difference among three major sign/symptoms ( Table 3).

Findings of post-treatment clinical outcome was associated with age. Time to recover from HFMD varied with child’s age. More young children (<5 years) recovered in <5 days (63/74, 85%) than older peers (≥5 years) (47/69, 69%) who were more likely to recover in >5 days) (P<0.05). However, clinical disease/outcome was not associated with children’s sex, although boys were more likely to suffer with the illness for 6–7 days, whereas girls tended to recover within 5 days. However, this was only marginally significant (P<0.05) ( Table 4).

**Table 4.** Composite table showing association of HFMD clinical features with season/local climate.

Variables	Post-treatment clinical outcome of childhood HFMD like-disease	
	Cured in >5 days (n=69)	Cured in <5 days (n=74)
Age of children (Mean= 2.9 ± 2.3 years)		
<1–3 years (n=78)	32	46
3.1–5 yeas (n=32)	15	17
5.1–10 years (n=33)	22	11
Chi-square ( *χ* ^2^) test:	p <.04	
Correlations	p <.02	
Sex		
Male (n= 80)	44	36
Female (n=63)	25	38
Fisher’s exact test	p <0.09 (2-sided), p<0.05 (1-sided)	
Pearson’s correlation	p <.07	

Complete raw data from each child assessed as part of this study Click here for additional data file. Copyright: © 2019 Hossain Khan MA et al. 2019 Data associated with the article are available under the terms of the Creative Commons Zero "No rights reserved" data waiver (CC0 1.0 Public domain dedication).

## Discussion

### Basis of this rapid appraisal on HFMD outbreak

Clinico-epidemiological insights from an extensive review on latest literature on HFMD augmented by our careful clinical observations on unusual events of febrile-rash (following WHO’s “Clinical management and public health response for HFMD” ^1^) made us enabled to establish the primary clinical diagnosis of childhood HFMD (M Azraf H Khan: Personal Observations, June–July 2017). Further, concurrent agreement from similar reports attested our diagnosis of HFMD in children, as correct ^1– 3^.

Gauging the potential of a sudden upsurge in HFMD cases in children (during July 2017) attending PMC-GH from its catchment area made us aware on an upcoming localized outbreak. A strategic plan was thus urgently adopted to conduct this rapid appraisal (short-term standardized surveillance) ^1^ on childhood HFMD utilizing a pre-set case-definition/syndromic approach based on WHO’s HFMD guidelines as depicted in Figure 4 (having fever or a history of fever, papulo-vesicular rash in extremities with or without oral ulcers), similar to a study conducted in Thailand ^29^.

The principal objective of this study (rapid appraisal) was to combat the impending HFMD outbreak with a secondary aim of disseminating the existence of newly emerged disease, thus to create a mass awareness. We also aimed to stir-up the local public health emergency squad to cumulate further strength towards combating such upcoming outbreaks in future. Finally, we desired to gauge strength of administrative drive, technical knowhow and clinical skill of PMC-GH team in combating that HFMD cases based on strong yet rational suspicions, as reported by others ^12, 13, 23^.

Keys to success of combatting that on-going pocket outbreak, were: **i)** Sincerity and devotion of PMC-GH team despite huge limitation in manpower and resources, **ii)** strong clinical eye suspecting HFMD as appropriate diagnosis ^12, 13, 23^ **iii)** instituting supportive therapy instantly, and **iv)** diagnosing HFMD despite gross lack in diagnostic facilities, though it often remains not essential in such emergencies ^14, 19, 25^.

### Potentials and dynamics of HFMD outbreaks

HFMD, emerged as a major public health problem in recent years ^2, 10^ was first recognized in the Western world ^1, 2^ during mid-1970s ^2^. It was then, spread out in Asia-Pacific region since mid-1990s ^3, 6, 9^, mostly in Malaysia, Taiwan, China and Singapore ^3, 6, 9, 11^. Though a longer time series is required to ascertain EV71 outbreaks of HFMD, it generally occurs in 2-to 3-year cyclical pattern in West Pacific Region (WHO/WPRO, 2010 ^9^) as reported from Singapore, UK, Malaysia and Japan ^9^. However, HFMD CA16 outbreak in Singapore also occurred periodically: in 2002, 2005 and 2007 but in 2006 but it was caused by EV71 ^11^. HFMD outbreaks were also reported from Orissa ^12^ and Calcutta ^13^ in India that borders with Bangladesh but strange is no published data or report exist in Bangladesh, yet (until June 2018) ^23^. In China, incidence and mortality were reported to be the highest among 12–23 months-old children ^34^.

All these facts and figures, including epidemiological hunches and variabilities support our strong speculation of this localized outbreak of HFMD in Pabna that we could combat successfully. We also postulate that HFMD might have emerged in Bangladesh earlier, but, swept on unnoticed being ‘underestimated’ due to its benign nature and self-limiting features ^6, 8^, or such latent HFMD cases or small localized outbreaks might remained under-reported or un-reported (Kazi Selim Anwar and Md. Abid Hossain Mollah, personal observation, June 2017). Our postulations partly remain similar to that of Xing *et al.* from China ^34^.

### Clinico-epidemiological perspectives

Using observation (clinical course, disease progression and outcome) re-confirms other reports that childhood-HFMD remains a benign and self-limiting disease ^6– 8^. We also attest that HFMD can be diagnosed accurately on physician’s strong rational suspicion ^13, 14^ and presenting signs/symptoms that can aid as sole diagnostic modality ^12^.

 In addition to history, onset and presenting clinical features ^6, 19^ we considered child’s socio-demographic characteristics ^12, 14^, and a positive history of similar sign/symptoms in child’s family, nursery/schools.

Our data yielded a significant association between age groups and three major clinical signs/symptoms. Moderate-to-high fever, painful oral ulcer and itchy-painful rash were more common in younger children- which remain consistent with other findings ^4– 6, 8, 9^. Moderate-to-high fever remains an important, but not mandatory or principal sign of HFMD, as the WHO’s guidelines for clinical and public health response indicate ^1^, in agreement with our findings. Oral and/or axillary temperature in 64% of cases revealed a moderate fever (38.5°C), ranging mostly between 37.5°C and 38.2°C; the rest (36%) had no or a low-grade fever (ranging between 37.0 to 38.4°C). These observations resembles with Van Pham *et al.* ^8^ though others reported high fever in HFMD-cases ^5, 9^.

**Figure 1.**
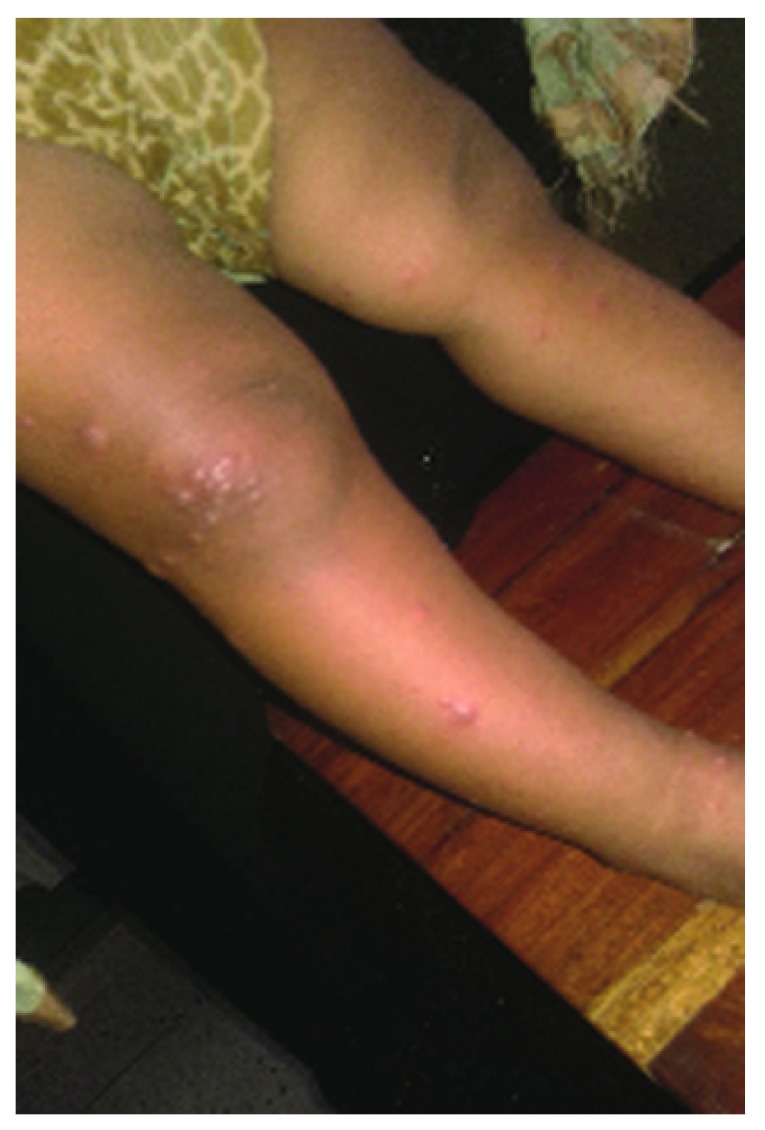
Multiple vesicular lesions containing turbid fluid seen in right knee of 4-year old girl.

Literature reveals papulo-vesicular rash as the most important characteristic symptoms for HFMD ^1, 4, 6, 14^ often manifesting as painful chicken-pox-like rashes in 60% cases ( Figure 3) though the rest 40% had it less painful or painless. Our findings on itchy rashes remain consistent with others ^1, 4^, particularly in its distributions (knees and/or buttock) ^1, 2, 7, 13, 14, 17^. Itchy rashe in child’s extremities that formed small pustules were filled with turbid fluid ( Figure 1) and in some cases it crusted off consequently ^6^ after 3–4 days- as other reported ^1, 4, 13, 14^, as well

**Figure 2.**
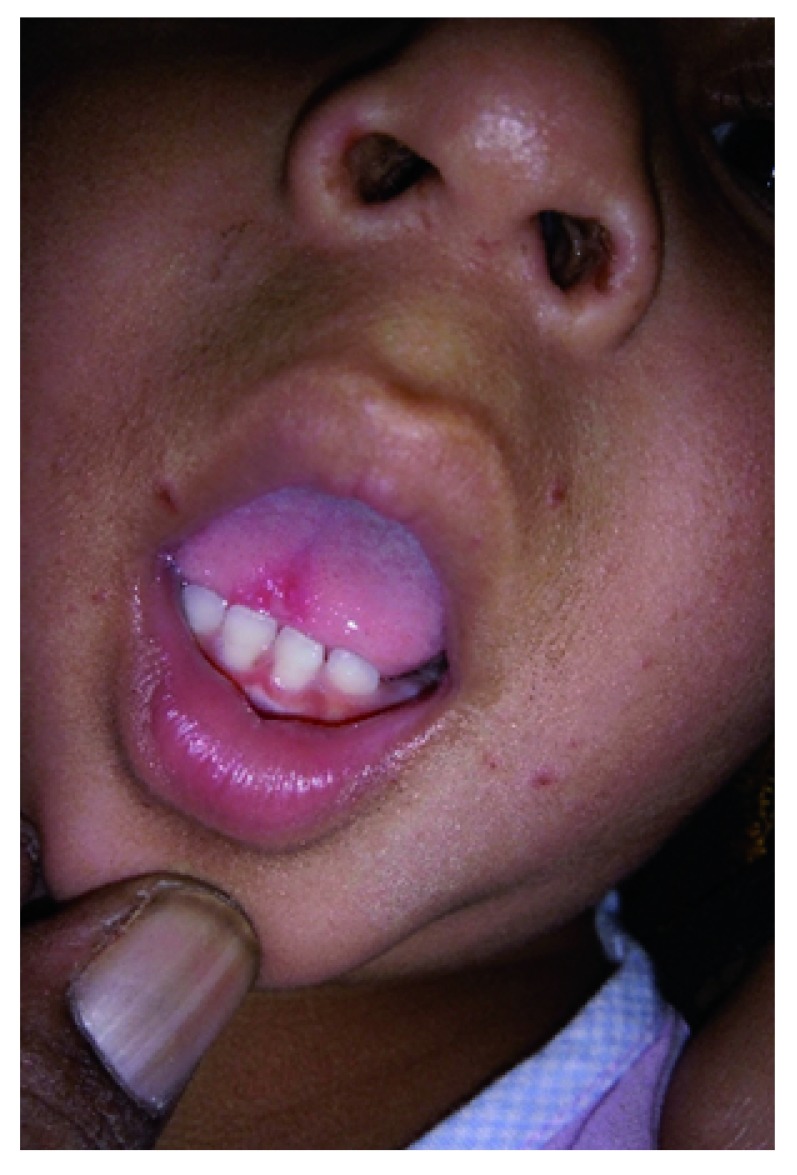
An oral ulcer on tongue with surrounding erythema of a 5-year old boy.

Secondly, most of the children (78%) had characteristic oral ulcers and/or painful blisters in tongue/mouth ( Figure 2), that remain similar to several reports ^1, 4, 7, 14, 16, 17^. However, the exact reason of less pain or painless oral ulcers/blisters in 22% cases in our study remain unclear. We guess it could be due to a varied perception and/or different tolerance, unwilling to mention, feeling shy or even being scared. Some of them may have taken analgesics at home prior to attending the hospital which they did not disclose despite repeated probing. Notably, sex of HFMD cases did not significantly differ with any sign/symptom except oral ulcers. More boys had it less painful than girls. Although a study in India reported an overall male-female ratio of 21:17 ^13^.

**Figure 3.**
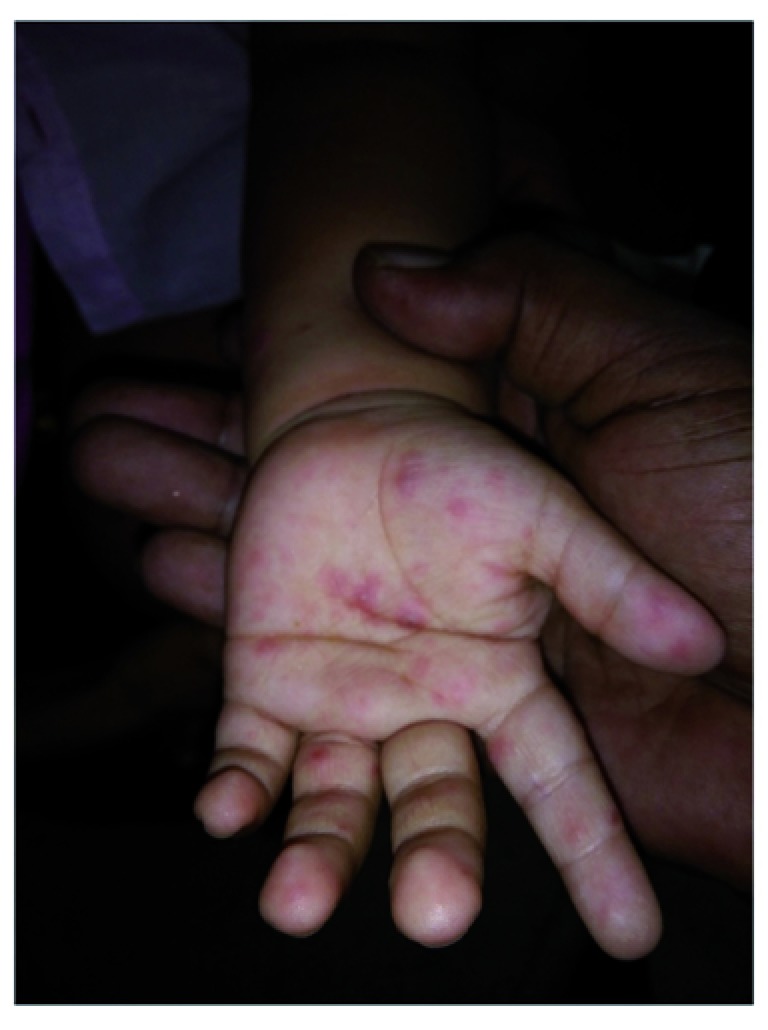
Papulo-vesicular lesions surrounded by erythematous zones on the left palm of a 1.5-year-old boy.

**Figure 4.**
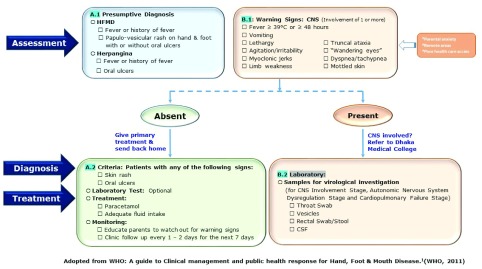
Decision tree for the clinical diagnosis and management of hand, foot and mouth disease.

### Differential Diagnosis of HFMD

Stringently examined thorough DD was performed to differentiate HFMD from closely similar diseases, like chicken-pox/varicella, scabies, measles, erythema multiforme, herpangina, herpetic gingivitis, drug eruptions, as several reports mentioned ^1, 6– 8, 22^. Mosquito bite was also included as report from India, underlined it as a simple yet valuable DD-point ^13^. Particular attention in the DD was paid on examining the characteristics of skin lesions (macules and papules quickly evolve into small vesicles manifesting on their palms, soles, and buttocks ^22^). We observed small vesicles in majority of these children that ruptured with the formation of erosions and crusts as ascribed by Sharma *et al.* Alike his finding, we also observed those vesicles as 1–5 mm in size as erythematous maculopapular lesions that rapidly enlarged by 3–15 mm progressing to vesicular eruption with prominent erythematous halo ^13, 14^ being comparable to that of a report by Bhumesh *et al.* from India ^21^.

### Laboratory diagnosis for HFMD

Laboratory diagnosis often remain unnecessary ^19^ to establish a diagnosis of HFMD. Use of WHO clinical case definition and exclusion of other similar syndromes through a stringent differential diagnosis usually remains adequate in most cases ^12, 23^. Moreover laboratory tests, such as serotyping, molecular, PCR and genotyping ^3^ and virus culture ^1, 13, 24^, may not be feasible, available and more importantly not affordable in resource-constrained countries ^12, 13^ like Bangladesh, particularly in hard-to-reach/remote areas. Although few studies report high WBC count or blood glucose, as associated with HFMD severity ^13– 19, 23, 24^, it remains scarcely seen in recent literature.

Virological assays remains the main diagnostic tool. Of the four species in the family of Picornaviridae (groups EV-A, B, C and D) that cause HFMD in children, EV 71 remain the most, followed by coxsackie-virus A6, A10, A16 ^3, 7, 8, 10, 24^. All these viruses are transmitted rapidly ^15, 17^ through direct contact, respiratory droplets, via feces/blister-fluid and through contaminated environment ^18^.

### Specific treatment for HFMD viruses

There is no specific treatment ^22, 25^ or pharmacological intervention ^4^ available for HFMD yet. Since it largely remain supportive ^19, 25^ we prepared a standardized therapeutic guide that was followed to take all therapeutic measures against HFMD cases. It consisted of: i) antipyretic/analgesics, ii) antihistamines, and iii) anesthetic gel or ointment. Since, skin lesion in these cases usually got healed within 3–4 days; we did not prescribe any acyclovir due to its reported adverse effects (nephropathy & neurotoxicity). Since oral acyclovir is poorly absorbed, we had to prescribe it for 5 days only in 8 severe cases (mean age, 2.4 years), exceptionally, in recommended dosage of oral syrup (20 mg/kg body-weight). But we found them (with profuse skin-lesions with severe pain) to respond to it dramatically, with early recovery. Reasons or basis of pathogenicity or pharmaco- dynamics is not fully understood demanding further investigations.

### Vaccination of HFMD

Though no effective vaccine available yet against HFMD viruses ^1, 2, 7^ scientists have been attempting to develop it in Malaysia (since 2010) ^33^, in China (since 2012) ^35^, and in Taiwan (since 2014) ^36, 37^. Cai *et al.* demonstrated how active immunization with an experimental inactivated CA16 vaccine can confer full protection by developing inactivated whole-virus vaccines against CA16 infection in human ^35^. Similarly, Chih-Wei Lin *et al*. ^36^ dissected ‘prospect & challenges’ with critical bottlenecks of developing multivalent HFMD vaccines. They demonstrated that combined vaccine will reduce number of shots that will simplify WHOs ongoing child immunization schedule, along with protecting kids from several viruses, viz., H5N1, EV71 & JEV at the same time ^36^. Yican Cui *et al.* attempted to develop a combined bivalent-vaccine comprising EV71 and A16 for receiving a balanced protective immunity ^37^ along with other developments in developing multivalent vaccines for broader protection for HFMD ^37^.

### Clinical outcome

Our clinico-epidemiological data, in agreement with other reports ^4, 6^ revealed that younger children (<5 years old) recovered quickly (in <5 days) than their elder peers (>5 years old) who recovered in 6–7 days (>5 days). This was similar to a report from China ^34^, too. There was a marginal significant difference in sexes: boys had seemingly quicker recovery than girls (P<0.05). However, among Chinese children: boys had FHMD in 1.6 times more than the girls ^34^. Nonetheless, latest literature attest most HFMD-cases recover within 7–10 days ^5, 8, 9^. These findings remain consistent with that of others from Asia-pacific countries ^4– 8^, including India ^12– 14, 18^.

### Complications

Though complications of childhood HFMD remain few, younger children may develop it more often ^7, 17, 21^. In our study, three cases (2.09%) developed complications of mild to moderate severity requiring special care. The first case (a 4-year-old girl) developed pneumonia requiring I/V antibiotics & was discharged following recovery after 2 days. The second one was an admitted case of pyoderma (a 5-year-old boy), who received appropriate antibiotics and was discharged after 3 days. The 3 ^rd^ case, a 1.5 year old girl having post-HFMD Onychomadesis ^38^ who were clinically diagnosed HFMD 25 days before. She had shedding of skin (right little finger) since few days. On repeated observations (weekly) her nail resumed in original position within 3 weeks without any medication. This scenario remains comparative to that of a report from South Korea ^38^. However, mechanisms of Onychomadesis and its association with HFMD is not yet fully understood as literature reveals ^38^ and that some viruses are responsible for onychomadesis as a temporal variation.

Although CA16 and EV71 ^2^ are mostly associated with neuro- respiratory syndromes ^1, 4^ we did not observe any of such severe diseases or serious complications, alike Vietnam study reporting 8.5% of severe cases ^8^, nor we encountered any death in our HFMD cases- a finding that remain consistent with several reports ^5, 6, 9, 15^.

### HFMD cases and local weather/climate

Several studies carried out in the Asia-Pacific region reporting an association of HFMD cases with a wide range of meteorological findings (weather, climate, ambient temperature, humidity, rain, etc.) ^5– 10, 15, 17, 18^. Reports on meteorological factors showing an association with HFMD outbreaks are: Singapore ^9, 15^, China ^10^ and Hong Kong ^20^. Mostly, rainy season ^8^ and short-term temperature variations ^20, 28^ had an impact on HFMD occurrence ^5^ in this region. This includes atmospheric pressure, relative humidity and rain precipitation- ^9^ that peaks in summer and in autumn ^18^ partly remain similar to that of ours. We observed this pocket outbreak of HFMD among children in early autumn (September to mid-October) and in late autumn/fall (mid-October to November), 2018. Interestingly, while in North China HFMD peaks in June, it is more in May and September-October southern China ^28^, which nearly corroborated with our findings ^34^.

One limitation is we could not conduct a proper meteorological study as reported from some Asian countries ^9, 10, 15, 20^. Contrarily, we only tried to find out briefly if local weather has any impact on HFMD just to acquire a preliminary idea in this aspect. A report from China also remain alike ours that seasonal patterns were weakly associated with climate and demographic factors ^34^.

However, the literature did not reveal any such study/report detailing the symptom-specific association of HFMD with seasons that we did, though some of our findings remain comparable with that of others ^5, 6, 9, 10^. Thus, data from this rapid appraisal (short-term surveillance) demonstrated certain seasonal characteristics of local weather were associated with fever and rash characteristics. Moderate-to-high fever was observed more often in fall/late-autumn (mid- October to November) than in early autumn (September to mid-October). We found similar result for rashes that predominantly occurred in fall than in early autumn. We did not observe an impact of rainfall/precipitation or ambient temperature on any of the 3 major signs/symptoms that we evaluated. We observed that childhood HFMD cases occurred mostly in dry weather with no rainfall (0.0 mm) almost equally in all three major signs/symptoms of HFMD including disease severity. These findings on local climatic factors did not corroborate with others ^5, 9, 10^.

### Socio-demographic characteristics and household economy of child’s family

Another unique strength of our study was to associate socio-demographic &/or HH-economy with child’s family with HFMD. Child’s age (mean ± SD, 2.9± 2.3 years) group remained similar to other reports ^1, 2, 7, 8, 14– 21^ Child’s age did not differ, significantly with sex. The HH structure revealed an average size of children’s family as 5.5±0.7 persons/HH, 62% of who were the first kids and 38% the second ones. Following World Bank categorized family/HH income /grades ^31^ majority of children’s family (85%) belonged to middle-income HHs living on a modest budget: 34.3% being in upper and 51% in lower, mid-income HHs. The rest 14.7% belonging to low-income HH are compelled to live with a very tight HH budget. Notably, a logical but unique finding, based on ecology, environment and health care utilizations, we observed HFMD cases more among first siblings than their siblings and who used to live in tight/low HH-budget. Of multifaceted reasons for this, we postulate that gross limitation in health care expenditure, distance of PMC-GH from HHs and low level of HH-income remains the major reasons. While it demands further explorations, few reports associating HFMD cases with personal hygiene and surrounding environment ^18, 26, 27^ remains important to stop transmitting HFMD-virus among adjacent communities.

### Prevention and control measures for HFMD

Due to a lack of available vaccines against HFMD-viruses ^15, 17, 18^ preventive measures remain the primary tool to circumvent HFMD-virus. Preventive methods include good personal hygiene, proper hand washing ^26^ particularly the post-defecation hand wash, pollution free environment ^12, 18^ and hygienic sewage management ^27^, ensuring germ-free drinking water and food ^18^. Although avoid person-to-person contact ^2^ through isolation remain justified, it often may not be practical in unprivileged communities of low-income countries having resource-constrained healthcare budget like Bangladesh. But it remains imperative that mass awareness be increased both among the communities and physicians.

### Insights on principal findings

• Our clinico-epidemiological observation indicates that childhood-HFMD has emerged in Bangladesh. Earlier outbreaks in Calcutta, India (bordering with Bangladesh) remains indicative of its introduction in this country since few years but remained unreported and thus, unnoticed.
• The physicians’ **strong yet** rationally judged clinical suspicion (based on signs/ symptoms) could establish a correct diagnosis, of HFMD cases.
• Stringent differential diagnosis remain indispensable to exclude similar fever- or rash-causing illness.
• Laboratory diagnosis seems unessential, particularly during HFMD outbreak situations when proper laboratory- diagnosis (virus culture, serology, molecular analysis) is not readily available.
• We experienced that early forecasting may aid in combating HFMD outbreaks in catchment areas to curb complications more successfully.
• Small-scale/localized outbreaks can be combated utilizing existing health-care/hospital set up/facilities.
• No specific treatment for HFMD exists, although supportive therapy can treat cases of HFMD in a week.
• Healthcare workers must remain aware on the prevention and treatment of HFMD, and, particularly on the warning signs of its severe illness.
• It is imperative to increase mass awareness to stop trans-mission of HFMD viruses (air/droplet, environment).
• Personal hygiene, hand washing and a pollution-free environment are mainstays of HFMD prevention

## Conclusion

We could diagnose cases of childhood HFMD successfully based on clinical signs/symptoms only and all cases recovered well within a week. Stringent differential diagnosis on similar rash and/or fever diseases/syndromes were deemed indispensable. The local climate may influence HFMD. Time consuming and costly laboratory diagnosis (virological/molecular) is not essential in resource-constrained settings, particularly during outbreak situations. No specific treatments or effective vaccinations exist for this often-underestimated disease yet. Supportive therapy and strict preventive measures is able to circumvent/destroy EV or CA viruses to combat ongoing HFMD-outbreaks/threats.

## Recommendations

Development of a globally representative multivalent HFMD vaccine remains necessary, particularly in countries where HFMD widespread, before it becomes pandemic. Both the government health services and meteorology departments should work together since climate is shown to be an early indicator of potential HFMD outbreaks. Our findings warrant that the countrywide public health emergency operations teams be more alert towards the effective prevention and control of HFMD in resource-constrained countries like Bangladesh. The governments of such countries should come up with a well-designed, sustainable strategic plan to combat upcoming HFMD outbreaks, in close-cooperation with national and global NGOs and UN organs to prevent its pandemic threat in the near future.

## Data availability

The data referenced by this article are under copyright with the following copyright statement: Copyright: © 2019 Hossain Khan MA et al.

Data associated with the article are available under the terms of the Creative Commons Zero "No rights reserved" data waiver (CC0 1.0 Public domain dedication). 

**Dataset 1. Complete raw data from each child assessed as part of this study.** DOI: 10.5256/f1000research.15170.d211038 ^39^.

## Consent

Written informed consent was obtained from the parents/guardians of each child for the publication of this report and the images contained within it.

## Author information

Kazi Selim Anwar is currently a Faculty at Department of Infectious Diseases, School of Medicine, International University of Health & Welfare (IUHW), Chiba, Japan.

## Supplementary material

**Supplementary File 1**

STROBE checklist
