# Supplementary material for: Emerging Hand Foot Mouth Disease in Bangladeshi Children- First Report of Rapid Appraisal on Pocket Outbreak: Clinico-epidemiological Perspective Implicating Public Health Emergency
Source: F1000Res. 2019 Jun 28;7:1156. Originally published 2018 Jul 30. [Version 3] doi: 10.12688/f1000research.15170.3 (PMC6662677; doi:10.12688/f1000research.15170.3)
Supplement: Peer review history

# Peer review: Reviewer response for version 3

**DOI:** 10.5256/f1000research.21485.r50588

**Published:** 2019-07-26

**Authors:** H Rogier van Doorn

**Affiliations:** 1 Oxford University Clinical Research Unit, Hanoi, Vietnam; 2 Centre for Tropical Medicine and Global Health, Nuffield Department of Clinical Medicine, Unversity of Oxford, Oxford, UK

The introduction is currently almost entirely factually correct, but could do with editorial review of the English used. This is beyond the responsibilities of this reviewer.

 Specific remaining comments:

 Pain assessment: I don’t understand the word ‘nullifying’ in the first bullet

 Demographic information: without knowing the distribution of SE groups in the catchment area of the hospital, what does this information on income groups add?

 Assessment of symptoms, 2 ^nd^ paragraph: “None of three… except fever and rash” Rephrase this

 The first two sections of the discussion can be shortened considerably, and perhaps moved to the introduction?

 Laboratory diagnosis of HFMD: Diagnosis remains necessary to assess quickly whether an outbreak is EV-A71 associated, which would warrant stricter prevention and mitigation measures

 The paragraphs on treatment and vaccination can be deleted. Not sure why antihistamines would be required.

 HFMD cases and local weather/climate: this section is too long and speculative.

I confirm that I have read this submission and believe that I have an appropriate level of expertise to confirm that it is of an acceptable scientific standard.

# Peer review: Reviewer response for version 3

**DOI:** 10.5256/f1000research.21485.r50587

**Published:** 2019-07-08

**Authors:** Md. Azizul Haque

**Affiliations:** 1 Department of Medicine, Rajshahi Medical College Hospital, Rajshahi, Bangladesh

I confirm that I have read this submission and believe that I have an appropriate level of expertise to confirm that it is of an acceptable scientific standard.

# Response

**Published:** 2019-07-11

**Authors:** Kazi Selim Anwar

**Affiliations:** Dept. of Infectious Diseases, International University of Health & Welfare (IUHW), Natalia Campus, Kozunomori, Narita, Japan

I, on behalf of all authors cordially thank reviewer-2 for reviewing our on-line published paper so brilliantly.  

We do extend all our best wishes for reviewing our paper with so much patience and upmost care with largely valuable comments and section/topic specific valuable suggestions being seriously careful towards adding values to improve the quality of our paper.

We cordially wish that any reviewer should possess such qualities, which we believe most reviewers do have those (except few).

We also recommend that the F1000Research should take up this reviewer (the 2nd one) in their reviewing panel if can not be taken in the editorial board right now.

We wish all the bests for our 2nd reviewer, per se.

# Peer review: Reviewer response for version 2

**DOI:** 10.5256/f1000research.18298.r48043

**Published:** 2019-05-22

**Authors:** Md. Azizul Haque

**Affiliations:** 1 Department of Medicine, Rajshahi Medical College Hospital, Rajshahi, Bangladesh

This is the first reported outbreak of HFMD from Bangladesh. This article will add some valuable information about distribution and burden of HFMD. Following observations has been made regarding this manuscript:

1. In abstract (background): “We report a localized outbreak of childhood HFMD for the first time from Bangladesh, diagnosed only based on clinical features due to gross lack in laboratory-diagnostic facilities.” The word “gross” should be removed from this sentence.
2. In abstract (results): please clarify and rewrite this sentence “Age did not differ with sex (P=0.98)
3. In Introduction “Severe cases occur red with a low incidence (3.2% to 8.5%) and fatalities are rare”, the word  “red” should be replaced by “rarely”
4. In results (demographic information), the sentence “Notably, children from mid-income-HHs contracted significantly more HFMD which was more among the first siblings (P<0.01)” should be rewritten as “HFMD cases were significantly more common among children from mid-income households and in the first siblings (p <0.001)
5. In results (assessment of symptoms) in the sentence “Similarly, papulo-vascular rashes weremore common in fall…” papulovascular should be replaced with papulo-vesicular.
6. The sentence should be replaced by “The three major sign/symptoms of HFMD were more prevalent on days where 0.0 mm precipitation was recorded.”
7. In the same section “Time to recover from HFMD varies with age of the patient”. In the sentence “More young children (<5 years) recovered in <5 days (63/74, 85%) than older peers (≥5 years) (47/69, 69%) who were more likely to recover in >5 days) (P<0.05).
8. In results (Clinico-epidemiological perspectives) section, the sentence “However, we neither observed such high incidences of sever disease alike from Vietnam (8.5%) nor recorded any fatal case in like others reporting as ‘none’ or ‘rare’ looks out of context and should be removed altogether or moved to complications section. If moved to the complications section, this sentence should also be rewritten as        

“In our series, we neither observed high incidence of severe disease, nor recorded any fatality.” Then comparison with data from other countries may be done.

  

1. While discussing severe disease and death, citation from Xing et al should be included, as his group published the largest epidemiologic study to date and showed the rate of severe disease and death in patients affected by HFMD (Xing W., Liao Q., Viboud C. Hand, foot, and mouth disease in China, 2008-12: an epidemiological study ^1^)
2. In results (Clinico-epidemiological perspectives) section, the sentence “Moderate-to-high fever, painful oral ulcer and itchy-painful rash were directly proportional to younger children which remain consistent with other findings” should be rewritten as “Moderate-to-high fever, painful oral ulcer and itchy, painful rash were more common in younger children; this finding is consistent with other studies”.
3. In results (Clinico-epidemiological perspectives) section, the sentence “This observation led us to postulate variation in body temperature led us to postulate that fever itself should not be considered as the sole symptom to confirm HFMD, rather this remain consistent with Van Pham *et al.*8 though others reported high fever in HFMD-cases5,9.” is redundant and should be removed altogether. This is unnecessary repetition of a statement made in the same para (Moderate-to-high fever remains an important, but not mandatory or principal sign of HFMD).
4. In results (Clinico-epidemiological perspectives) section, citation is needed for the sentence “Literature reveals papulo-vesicular rash as the most important characteristic symptoms for HFMD often manifesting as painful chicken-pox-like rashes in 60% cases (Figure 3) though the rest 40% had it less painful or painless.”
5. In results (Clinico-epidemiological perspectives) section, the sentence “Since pain remains subjective in younger children in expressing pain intensity, we categorized HFMD cases based on having no history of recent pain issues, facial expression of child on slight touch on rash/oral ulcer including mother’s impression plus clinician’s rational judgement.” is redundant and should be removed altogether. Pain assessment/scoring tool has been described in detail in the Methods section.
6. In results (Clinico-epidemiological perspectives) section, the sentence “However, the exact reason in 22% less pain or painless oral ulcers/blisters remain unclear.” should be rewritten as “However, the exact reason of less pain/painless oral ulcers/blisters in 22% cases in our study remain unclear.”  
7. In Laboratory Diagnosis for HFMD, the sentences “Laboratory diagnosis is usually not essential12,23 to confirm a readily diagnosed HFMD case based on rational judgement of existing clinical features. Even, lab diagnosis often remains unnecessary19” should be rewritten as “Laboratory diagnosis often remain unnecessary to make a diagnosis of HFMD. Use of WHO clinical case definition and exclusion of differential diagnosis is adequate in most cases.”
8. Reference number 12 may be omitted as authors of that article used RT PCR for virus isolation in 7 cases.
9. The para “Furthermore, raised blood glucose level may be due to other viral diseases rather than HFMD, and may confound by other infections and/or inflammatory processes. Moreover, drawing intravenous blood from younger children possessing thin veins may be extremely difficult if not possible particularly in primary care health centers in grass-root level. Children often exhibits agitation when attempts are made to draw blood showing grossly non-compliance and non-cooperative” is redundant and should be removed.
10. In complications, the sentence “Our finding yielded three cases (2.09%) complications of mild-to-moderate severity who we had to pay particular attention to” should be rewritten as “In our study, three cases (2.09%) developed complications of mild to moderate severity requiring special care”. 

I confirm that I have read this submission and believe that I have an appropriate level of expertise to confirm that it is of an acceptable scientific standard.

## References

[1] : Hand, foot, and mouth disease in China, 2008–12: an epidemiological study. *The Lancet Infectious Diseases*.2014;14(4) : 10.1016/S1473-3099(13)70342-6 308-318 10.1016/S1473-3099(13)70342-6 PMC403501524485991

# Response

**Published:** 2019-05-23

**Authors:** Kazi Selim Anwar

**Affiliations:** Dept. of Infectious Diseases, International University of Health & Welfare (IUHW), Natalia Campus, Kozunomori, Narita, Japan

I, on behalf of all authors whole heatedly thank the reviewer for reviewing our online published paper with so much care and attention, yet so productively and meaningfully. We, the authors, remain totally satisfied on the way the reviewer looked into every bits of our paper & in so in-depth, yet so positively.  

**Comment: **Yes we agree to all of the the well-chalked & thoughtful queries, along with all his suggested points to bring some minor changes in our paper, soon.  

**Final comment for the Reviewer** *(only if the F1000Research allow me to do so, please)* **:**

This is one of the best review I have had encountered ever so far (I am engaged in reviewing at least 6 globally reputed Scopus indexed journals having good impact factors).

May I, thus, recommend the F100Research group/authority to take this reviewer as one of its regular reviewers, like me, who would definitely add more significant values of International Board of Reviewers or Editorial Panel of F100Research. It would facilitate more wider scopes towards more standardized publishing, I believe, that the journal has been doing that always, of course! 

Dr. Kazi Selim Anwar, in favour of all authors of this online published paper.

# Peer review: Reviewer response for version 2

**DOI:** 10.5256/f1000research.18298.r40496

**Published:** 2018-12-04

**Authors:** H Rogier van Doorn

**Affiliations:** 1 Oxford University Clinical Research Unit, Hanoi, Vietnam; 2 Centre for Tropical Medicine and Global Health, Nuffield Department of Clinical Medicine, Unversity of Oxford, Oxford, UK

I thank the authors for addressing a number of my comments.

 I still think the discussion is too long and deals with a large of number of topics that are irrelevant to the data presented, being a clinically diagnosed first-time outbreak of HFMD in Bangladesh in a single hospital. 

 There is already a large body of available literature on HFMD. This manuscript adds the description of an outbreak or upsurge in Bangladesh. That is what needs to be described and interpreted / compared with other outbreaks.

 There is no need in this publication for the discussion section to provide a general review on HFMD or to provide extensive general recommendations on HFMD diagnosis, treatment and prevention.

 The fourth paragraph of the discussion is an interpretation for which the authors present no data, this shouldn't be in a scientific publication. Sections on laboratory diagnosis, specific treatment and vaccination are not needed in this report. The list of principal findings should also be limited to findings from THIS study. 

 In the introduction, rephrase the sentence on "pollution free environment". HFMD prevention is through avoiding contact with infected persons and their secretions and excretions through hygiene/hand-washing and distancing (e.g. school closure or sending sick kids home).

I confirm that I have read this submission and believe that I have an appropriate level of expertise to confirm that it is of an acceptable scientific standard, however I have significant reservations, as outlined above.

# Peer review: Reviewer response for version 1

**DOI:** 10.5256/f1000research.16525.r38927

**Published:** 2018-10-02

**Authors:** H Rogier van Doorn

**Affiliations:** 1 Oxford University Clinical Research Unit, Hanoi, Vietnam; 2 Centre for Tropical Medicine and Global Health, Nuffield Department of Clinical Medicine, Unversity of Oxford, Oxford, UK

The authors describe the results from a prospective observational study of children attending a single hospital in Bangladesh using WHO diagnostic criteria. If this is the first time HFMD has been described from Bangladesh, this is of major relevance locally and regionally.

 I recommend the authors to use the STROBE guidelines/checkbox to verify whether all required data are included.

 The main findings are that a number of cases of HFMD among young children was described from one hospital in Bangladesh, the symptom and age distribution are relatively similar to what is known from the region. Healthcare workers should be aware of this illness, its prevention and treatment and warning signs of severe illness.

 I have made some comments and suggestions below, the most important being to shorten and bring more focus on the current data in the discussion.

 Specific comments:

- Add this sentence on the aetiology, to replace the sentence in the second paragraph of the introduction: "HFMD is caused by several serotypes of Enterovirus A, the most common being Enterovirus A71 (EV-A71) and Coxsackievirus A16 (CV-A16) and more recently also CV-A6 and CV-A10. EV-A71 is associated with a higher proportion of severe illness."
- Please add the exact case definition that was used to enrol children.
- 143 children were included, how many children were eligible during the period of enrolment? How many were not enrolled because of exclusion criteria or otherwise, how many didn't consent?
- Can an epidemiological curve be added?
- Any further information on cases in the region, nearby hospitals?
- Were any warning signs detected during the study?
- Because of the epidemiology of HFMD, the preferred age stratification would be 0-6, 6-12, 12-24, 24-60 and >60 months or similar (e.g. Xing et al ^1^)
- It is common to study the effects of precipitation allowing for a lag period of few days (incubation period)
- Reviews on the epidemiology, mortality and long-term outcome of HFMD have been published recently. These can be referenced in the discussion for clarity.
- The discussion deals with a broad spectrum of general topics. This is appropriate for a report to be circulated among local healthcare workers, but not for the current scientific publication. I would suggest to focus on the data from the current study for the discussion here, to broadly describe the findings and if there were any striking differences with what has been described from the region. The authors should not overinterpret the data from this relatively small sample size to look for potential associations.
- In the third paragraph of the discussion the authors state that outbreaks occurred in 1997 and 1998, despite forecasts. The referenced forecasts were derived from timeseries from Malaysia from 1998-2006 and could not have predicted the 1997-8 outbreaks. There are syndromic and serotype specific timeseries from Japan dating back to the 1980s that may have forecasted these, but to my knowledge no major HFMD outbreaks had occurred in Malaysia and Taiwan prior to these.
- In the laboratory diagnosis section, it is important to realise that diagnosis of EV-A71 as the main pathogen is important as it is associated with a higher proportion of severe illness.

I confirm that I have read this submission and believe that I have an appropriate level of expertise to confirm that it is of an acceptable scientific standard, however I have significant reservations, as outlined above.

## References

[1] : Hand, foot, and mouth disease in China, 2008–12: an epidemiological study. *The Lancet Infectious Diseases*.2014;14(4) : 10.1016/S1473-3099(13)70342-6 308-318 10.1016/S1473-3099(13)70342-6 PMC403501524485991

# Response

**Published:** 2018-10-19

**Authors:** Kazi Selim Anwar

**Affiliations:** Dept. of Infectious Diseases, International University of Health & Welfare (IUHW), Natalia Campus, Kozunomori, Narita, Japan

**Comment specific response from the authors:**

The authors thankfully appreciate the reviewer-1 for **approving **the paper with reservations and thank for the kind review and comments.

**The followings remains the comment specific response from the authors:**

**Comment-1:** The authors describe results from a prospective observational study of children attending a single hospital in Bangladesh using WHO diagnostic criteria. If this is the first time HFMD has been described from Bangladesh, this is of major relevance locally and regionally.

**Reply-1: **Yes, this observational study in our hospital remains the first report on HFMD from Bangladesh. We, therefore, thank you sincerely, for such an important comment….  “it remains of major relevance locally and regionally”.

**Comment-2:** I recommend the authors to use STROBE guideline/checkbox to verify if all required data included

**Reply-2: **Yes, agreed. We have uploaded **STROBE** guideline to verify our data in revised version.

**Comment-3: **The main findings that a number of cases of HFMD in young children was described from one hospital in Bangladesh, symptom & age distribution are relatively similar to what is known from the region.

**Reply-3: **Yes, we are glad to see your valuable comment that HFMD symptoms in younger children remain relatively similar with that of other reports from South/SE- Asian region.

**Comment-4: **Healthcare workers should be aware of this illness, its prevention and treatment and warning signs of severe illness

**Reply-4: **Yes, we appreciate your comment that healthcare workers must be aware of HFMD its prevention & treatment, particularly on warning signs of severe illness. Mentioned in 8 ^th^ bullet point of “Insights on principal findings”.

**Comment-5: **I have made some comments and suggestions below, the most important being to shorten and bring more focus on the current data in the discussion.

**Reply-5: **Yes, agreeing to your most suggestion, shortened the discussion part focusing on current data-that really makes sense. Shortened discussion part as suggested

**Comment-6:** Add this sentence on the aetiology, to replace the sentence in 2nd para-graph of introduction **….**"HFMD is caused by several serotypes…. with a higher proportion of severe illness."

**Reply-6: **We thankfully agreed to replace it in the 2 ^nd^ paragraph of introduction.. HFMD is caused by several serotypes of enterovirus A, the most common being enterovirus A71 (EV A71) and coxsackievirus A16 (CV-A16) and more recently, also (CV A-6, and CV A-10). EV-A71 is associated with a higher proportion of severe illnesses "

**Comment-7.** Please add the exact case definition that was used to enroll children

**Reply-7:** Though it is mentioned in Fig.4, we have reemphasized the WHO recommended exact case definition of HFMD: having i) fever or history of fever, ii) papulovesicular rash on hand & foot iii) with or without oral ulcers.

Added this case definition in 2 ^nd^ line of method and in clinical diagnostic tool, as well.

**Comment 8:  **.… 143 children were included how many were eligible during enrolment period? How many were not enrolled because of exclusion criteria or otherwise, how many didn't consent?

**Reply-8: **Since it was an outbreak situation, we had to enroll all 143 children attending our hospital from Sept. to Nov., 2017 with suspected HFMD cases (who met WHO criteria). Guardians of all children provided written consent to enroll.

**Comment 9:** Can an epidemiological curve be added?

**Reply-9: **Well yes, but we have described almost all epidemiological features in tables.

**Comment 10:** Any further information on cases in the region, nearby hospitals?

**Reply-10:**  No. We explored to determine that among surrounding families, nurseries or kindergarten/primary schools, but none revealed any positive information.

**Comment 11: **Were any warning signs detected during the study

**Reply 11:** No, not as such. Of the 3 complications that we observed, only girl had onychomadesis, 1 child had pneumonia and the other had pyoderma. These may well be regarded as ‘cautionary’, if not ‘warning’ signs.

**Comment 12:** Because of the epidemiology of HFMD, the preferred age stratification would be 0-6, 6-12, 12-24, 24-60 and >60 months or similar (Xing et al ^1^)

**Reply 12: **Yes. But during that pocket outbreak our hospital team categorized the HFMD victimized children into two groups of <5 and >5 years only. Since 77% of them fell under <5 years it was further categorized into <3 years & 3.1 to <5 years. This age-stratification was done to fit aged-matched cases facilitating analysis.

**Reply 13: **It is common to study the effects of precipitation allowing for a lag period of few days (incubation period)

**Reply 13: **Yes. But we could not do that due to paying more attention in tackling/combating the on-going emergency of that pocket outbreak.

**Comment 14:** Reviews on the epidemiology, mortality and long-term outcome of HFMD have been published recently. These can be referenced in the discussion for clarity.

**Reply 14: **Well, yes. But we have described some of those in our discussion already.

**Comment 15:** The discussion deals with a broad spectrum of general topics. This is appropriate for a report to be circulated among local healthcare workers, but not for the current scientific publication.

I would suggest to focus on the data from the current study for the discussion here, to broadly describe the findings and if there were any striking differences with what has been described from the region. The authors should not overinterpret the data from this relatively small sample size to look for potential associations.

**Reply 15: **Thanks for the good suggestions. We have shortened the discussion part, focused on our data from our current study and tried to describe the striking findings only that yielded some regional differences. And we also tried to avoid over-interpreting our data (relatively small sample size).

**Comment 16: **In the third paragraph of the discussion the authors state that outbreaks occurred in 1997 and 1998, despite forecasts. The referenced forecasts were derived from time series from Malaysia from 1998-2006 and could not have predicted the 1997-8 outbreaks. There are syndromic and serotype specific time series from Japan dating back to the 1980s that may have forecasted these, but to my knowledge no major HFMD outbreaks had occurred in Malaysia & Taiwan prior to these.

**Reply 16: **Thanks for pointing it out rightly. After cross checking on the contents of this sentence we have removed the following sentences 'Despite epidemiological forecasts that HFMD outbreaks occur in a 2–3-year cyclical pattern two large epidemics broke out in 2 consecutively years: one in Malaysia during 1997 and the other in Taiwan, the following year." Corrected this part as edited in the 2 ^nd^ paragraph of ‘Potentials & dynamics of HFMD outbreak’.

**Comment 17:** In the laboratory diagnosis section, it is important to realise that diagnosis of EV-A71 as the main pathogen is important as it is associated with a higher proportion of severe illness.

**Reply 17: **Yes. Good point. We have added this point in lab diagnosis sect giving importance to diagnose EV-A71 as the main pathogen causing proportionately more severe cases of HFMD. It was reflected in 3 ^rd^ paragraph of laboratory diagnosis, properly.

Finally the authors thank the reviewer-1 for his kind comments and suggestions once again.
